# Prospective and Pavlovian mechanisms in aversive behaviour

**DOI:** 10.1016/j.cognition.2015.10.017

**Published:** 2016-01

**Authors:** Francesco Rigoli, Giovanni Pezzulo, Raymond J. Dolan

**Affiliations:** a Wellcome Trust Centre for Neuroimaging, University College of London, London, UK; b Institute of Cognitive Sciences and Technologies, National Research Council, Rome, Italy; c Max Planck UCL Centre for Computational Psychiatry and Ageing Research, London, UK

**Keywords:** Aversion, Pavlovian, Goal-directed, Controllability, Threat distance, Fear, Anxiety, Learned helplessness

## Abstract

Studying aversive behaviour is critical for understanding negative emotions and associated psychopathologies. However a comprehensive picture of the mechanisms underlying aversion is lacking, with associative learning theories focusing on Pavlovian reactions and decision-making theoretic approaches on prospective functions. We propose a computational model of aversion that combines goal-directed and Pavlovian forms of control into a unifying framework in which their relative importance is regulated by factors such as threat distance and controllability. Using simulations, we test whether the model can reproduce available empirical findings and discuss its relevance to understanding factors underlying negative emotions such as fear and anxiety. Furthermore, the specific method used to construct the model permits a natural mapping from its components to brain structure and function. Our model provides a basis for a unifying account of aversion that can guide empirical and interventional study contexts.

## 1 Introduction

Given their fundamental importance in evolution, the strategies adopted by living organisms to manage danger have been extensively studied. Early associative-learning theorists proposed that aversive behaviour is guided by simple instrumental principles prescribing that punishment diminishes the probability of performing an action while avoidance of, and relief from, punishment reinforces the probability of performing a similar action (Dinsmoor, 2001, Rescorla and Solomon, 1967, Solomon and Brush, 1956, Thorndike, 1911). Bolles (1970) criticised this framework arguing it was based on a wrong assumption that all actions in the animal’s repertoire have the same prior chance of being selected and instead argued that there are species-specific defensive reactions, selected by evolution, which are preferentially activated and replaced by other responses only after repeated punishments. This derived from particular observations, for example the fact that rats usually exhibit a specific freezing response to fearful stimuli and can learn only a small set of responses to avoid punishment, with each response requiring a certain amount of learning experience (Bolles, 1970).

More recent findings argue even more strongly against a central role for instrumental learning as they show that in some cases repeated experience of electric shock increases (rather than diminishing) the probability of performing a pre-specified response such as freezing (Fanselow & Lester, 1988). These data highlight the existence of a set of innate (i.e., Pavlovian) aversive reactions elicited by certain conditions of shock temporal delay, as rats froze immediately after the presentation of a conditioned stimulus, while just before and after a shock they exhibited a fight/flight reaction consistent in jumping, biting and vocalizing (Fendt & Fanselow, 1999). A similar response pattern was observed when manipulating the spatial, instead of temporal, threat distance, together with the observation that rats engage in cautious exploration (described as risk-assessment behaviour) when a threat is not actually present but is potential, such as in a novel context or where a predator has been previously seen (Blanchard & Blanchard, 1989).

Another important modulator of aversive behaviour is controllability. In a classic experiment on learned helplessness (Seligman & Maier, 1967), one group of dogs learnt to press a lever to terminate non-signalled electric shocks whereas a second group received shocks exactly contemporaneously to the first group but had no actual control on shock delivery, a procedure ensuring punishment was matched in terms of number, intensity and time across groups. After the learning phase, the two groups were tested in a new environment in which a jumping response could be learnt to avoid shocks. Here the dogs trained with controllable punishments learnt the instrumental safety response whereas the other group failed to learn this response. The finding is widely interpreted as indicative of a generalisation of uncontrollability beliefs from one context to the other (Maier & Seligman, 1976) or, alternatively, as due to the fact that uncontrollable punishments increase stereotypical fear responses (e.g., freezing) which interfere with the performance of alternative actions (Desiderato and Newman, 1971, Mineka et al., 1984).

Altogether, associative learning theories view aversive behaviour as determined by a set of stimulus–response associations, either shaped by experience (i.e., instrumental) or innate (i.e., Pavlovian), and modulated by temporal/spatial threat distance and controllability. A striking example of Pavlovian–instrumental interaction is negative auto-maintenance (Williams & Williams, 1969), in which pigeons trained with a light-food association exhibit a conditioned response of pecking the light even when, in a test phase, food is delivered solely as a consequence of non-responding. These and similar findings represent the building blocks of the idea that flexible instrumental mechanisms are activated together with rigid Pavlovian tendencies that usually facilitate performance but, given their rigidity, in some circumstances have maladaptive consequences (Dayan et al., 2006, Guitart-Masip et al., 2014, Moutoussis et al., 2008, Rigoli et al., 2012). However, several fundamental theoretical aspects remain to be clarified. First, in which conditions are instrumental rather than Pavlovian responses elicited? Second, what is the specific role of threat distance and controllability in modulating aversive behaviour? Third, dating back to Tolman’s notion of latent learning (1932), research in the appetitive domain has investigated a form of instrumental behaviour guided by goal-directed processes which are based on stimulus-action-outcome associations, but the part played by these mechanisms in the aversive domain remains unclear (Balleine and Dickinson, 1998, Dickinson and Balleine, 1994).

Here, we connect associative learning theories of aversion and theoretical models of the instrumental–Pavlovian interaction with a specific focus on goal-directed mechanisms. We propose that threat distance and perceived controllability modulate a goal-directed/Pavlovian relationship by increasing the weight one controller exerts over the other. Specifically, we argue that proximal threat distance and low controllability boost a Pavlovian weight, based on observations of increased freezing and fight/flight response (hallmarks of Pavlovian control) in this condition. Conversely, larger threat distance and higher controllability boost goal-directed mechanisms, a process we interpret as underlying risk-assessment behaviour observed in rodents under potential threats. We formalise these intuitions in a biologically plausible computational model and then test whether this model can reproduce reported empirical data.

## 2 A model of the goal-directed/Pavlovian interaction in aversion

We introduce a theoretical model whose aim is to describe the computational processes underlying the expression of aversive behaviour. We highlight a link to a set of neural network models that combine reinforcement learning principles within a biologically plausible implementation (e.g., Frank et al., 2004, Miller and Cohen, 2001, Reynolds and O’Reilly, 2009). An advantage of this model is that it can be linked to neurobiology given that each component is mapped to a specific neural structure or set of structures. The model rests on a distinction between goal-directed and Pavlovian control (Balleine and Dickinson, 1998, Dayan et al., 2006, Guitart-Masip et al., 2014, Rigoli et al., 2012), where each system uses a specific algorithm to compute an estimate of the expected value linked to a given context. The Pavlovian controller learns to associate expected values directly with stimuli, depending on stimulus-punishment contingencies, whereas the goal-directed controller learns to associate expected values with stimulus-action-outcome associations. Eventually each controller selects an action. For a given stimulus, the Pavlovian controller always chooses the same innate reaction, whereas the goal-directed system can flexibly choose different actions according to a softmax rule (Daw, O’Doherty, Dayan, Seymour, & Dolan, 2006). Finally, the innate Pavlovian response and the action selected by the goal-directed controller are activated proportionally to the weight of the corresponding controller, and these actions cooperate or compete depending on their compatibility. Threat distance and perceived controllability are the key variables that modulate the engagement of a controller. The influence of threat distance is represented as a boosting effect on goal-directed activation as a function of increasing distance. The role of perceived controllability is more complex as this variable is factorized into two subcomponents, the first dependent on controllability related to a specific stimulus and the second on a generalised belief independent of stimuli.

**Fig. 1:**
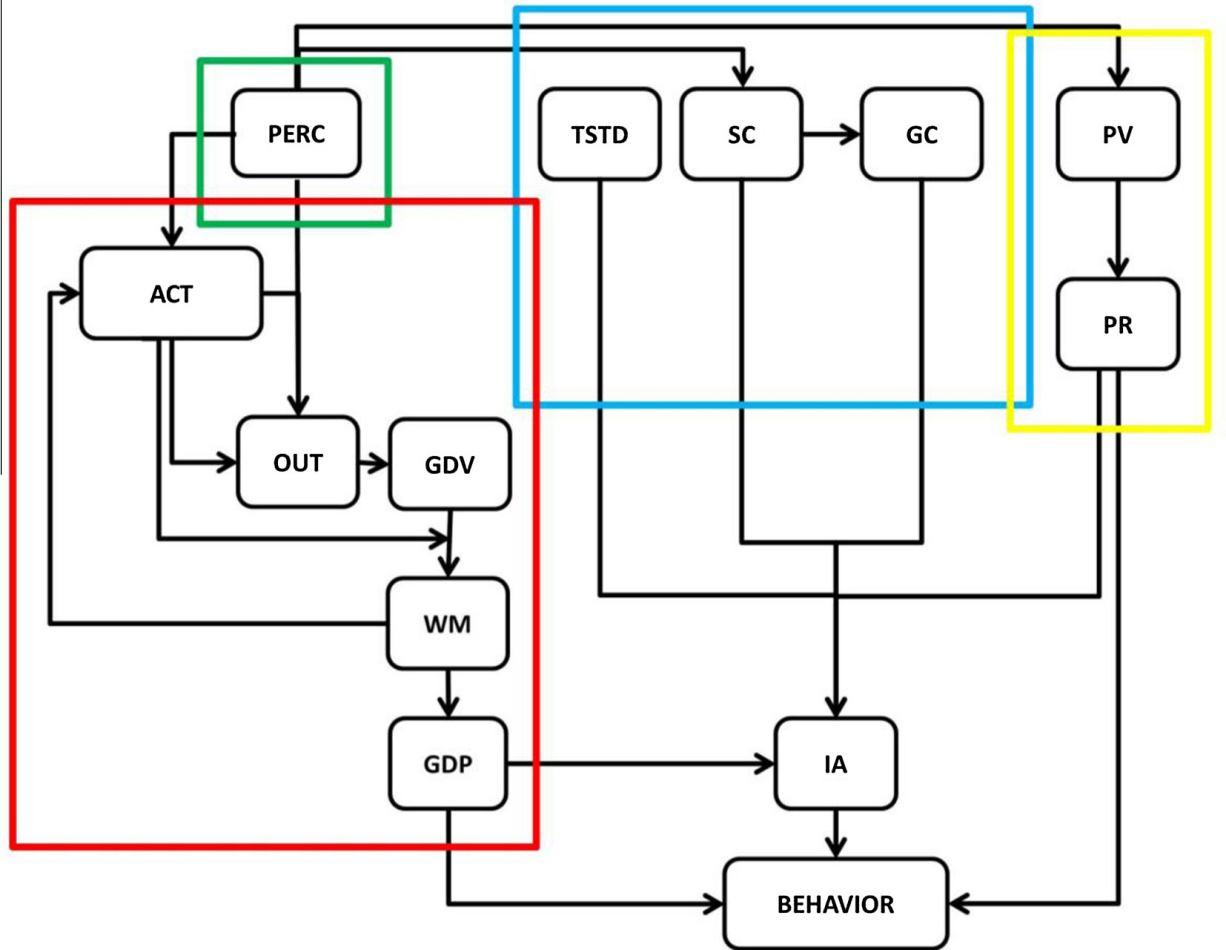
Architecture of the computational model. Coloured boxes indicate the subsystems (green: perceptive subsystem, red: goal-directed subsystem, yellow: Pavlovian subsystem, blue: modulatory subsystem) and black boxes represent the computational layers. Arrows indicate the connections among layers. PERC: perception; ACT: action; OUT: outcome; WM: working memory; GDV: goal-directed value; GDP: goal-directed plan; TSTD: temporal and spatial threat distance; SC: specific controllability; GC: general controllability; PV: Pavlovian value; PR: Pavlovian response; IA: instrumental ability.

More specifically (see Appendix A and Fig. 1), the model describes an agent’s computations during aversive conditions as emergent from different subsystems organised in layers each composed of different nodes. An input from the environment is represented as the activation of a specific node in a Perceptive layer (PERC). PERC activates a goal-directed subsystem composed of different layers, namely Action (ACT), Expected Outcome (OUT), Expected Goal-directed Value (GDV), Working Memory (WM) and Goal-directed Plan (GDP). ACT, representing the current simulated action during planning, encodes each action as activation of a specific node. PERC and ACT are connected to OUT, which represents likely future states of the world in which each node represents an expected outcome. A given combination of PERC and ACT activity corresponds to a specific input to OUT. Each OUT node activity, computed as the input value divided by the sum of all other inputs to OUT, can be conceived as the conditional probability of the corresponding expected outcome, given PERC and ACT activity. All OUT nodes are connected to GDV, which is computed as the sum of OUT node activities, each node multiplied by its expected value (encoded by the OUT–GDV connection weights). Once this value is computed, it is stored in WM which records the different action values.

The goal-directed subsystem follows a cyclic dynamic through which, once PERC is activated, an action simulation process is elicited consisting in sequential activation of different ACT nodes, and in the evaluation (encoded in GDV) of their likely consequences (encoded in OUT). More specifically, when a stimulus is presented, the first action in the repertoire is activated in ACT and this activates OUT and in turn GDV. WM encodes the expected value of the first action (corresponding to the activation of the first GDV node) and, through a recursive connection to ACT, inhibits the activation of the ACT node corresponding to the first action, eliciting activation of the second-action ACT node. Therefore, a new OUT and GDV activations are computed and the latter recorded in WM. When all actions have been simulated and the corresponding expected values recorded in WM, the goal-directed subsystem makes a choice. In keeping with human evidence (Daw, O’Doherty, Dayan, Seymour, & Dolan, 2006), action is chosen according to a softmax rule and the chosen action is coded as activation of a specific GDP node. The activated GDP node acquires the activation level of the higher activation WM node, even if the two nodes do not correspond to the same action.

So far the goal-directed subsystem is characterised within a one-step temporal horizon. Though in simulations we focus on this special case (see below), the model can be extended to more distant temporal horizons. However, in this case the goal-directed subsystem needs to evaluate policies, namely sequences of actions, rather than single actions alone. This is achieved by adding a number of ACT, OUT and GDV layers equal to the number of time steps the agent plans ahead, plus a policy (POL) and a GDV-SUM layer. Goal-directed planning works again in a recursive manner starting with activation of the first node of POL, which in turns switches on a specific combination of nodes within the different ACT layers along time. As before, activity in the first (in temporal order) ACT and in PERC results in a specific activation in the first OUT (in which each input is divided by the sum of all other inputs) and GDV. In a cascade process, activation in the first OUT and second ACT propagates to the second OUT up to the second GDV and so forth. Activations of all GDVs along time are summed up in GDV-SUM (note that a discount parameter can be implemented at this stage) and stored in WM, which, thanks to the same mechanism described above, inhibits the first POL node and activates the second POL node, for which the process is repeated. Eventually, all policies are simulated and the corresponding expected values are encoded within WM.

In parallel with recruiting the goal-directed system, PERC also triggers the Pavlovian subsystem, composed of a Pavlovian expected Value (PV) and Pavlovian Reaction (PR) layers. Every stimulus is associated with a specific PV activation, depending on the weights of the PERC–PV connection. In turn, PV activates PR that represents the innate conditioned or unconditioned motor response triggered by PERC and whose activation is proportional to PV.

PERC is also connected to a modulator subsystem representing controllability and threat distance. The former is implemented through two layers, namely Specific Controllability (SC) and Generalised Controllability (GC), and the latter corresponds to the Temporal and Spatial Threat Distance (TSTD) layer. For the implementation of controllability, we follow learned helplessness theory (Maier & Seligman, 1976) maintaining that the controllability associated with a specific context corresponds to the conditional probability of avoiding a punishment with the best action, minus the probability of avoiding the punishment without that action, multiplied by the value of that punishment. The first component (SC) represents controllability relative to a given context and simply corresponds to the difference between the maximum and minimum action values within the WM layer. The second component (GC) represents a more abstract variable which depends on past controllability experience independent of context. After each new trial, GC is updated according to a delta rule based on the SC value at that trial and independent of which stimulus is present. We hypothesise that GC is important to model learned helplessness effects by which animals, after repeated uncontrollable punishments, cannot learn an appropriate instrumental action in a novel context, an effect that could arise out of an uncontrollability bias developed after repeated experience (Huys & Dayan, 2009). Finally, in relation to threat distance, the corresponding TSTD activation corresponds to the time or space to the threat.

The different subsystems determine the behavioural output of the model as their activities are summed up in the so-called Instrumental Ability (IA) node, representing the activation of the goal-directed system. In particular, IA is positively correlated with GDP, SC, PR, GC and TSTD. Finally, a motor output (BEHAVIOUR) is computed based on a logistic regression of IA. The probability that BEHAVIOUR corresponds to GDP or PR is directly and inversely proportional to IA respectively.

So far, we have described the model structure and its decision processes. We now explain the model’s learning mechanisms. Once BEHAVIOUR is executed, an outcome (OUTCOME) is obtained in the environment and is used for learning. The weight of the PERC–ACT–OUT connection is updated based on Hebbian rules, in other words the link between the active PERC node, the ACT node corresponding to BEHAVIOUR, and the OUT node corresponding to OUTCOME is strengthened at each new experience. The connection between the OUT node corresponding to OUTCOME and GDV is modified following a temporal difference algorithm (Sutton & Barto, 1998) as well as the connection between the active PERC node and PV. GC is updated following a delta rule based on the value of SC in a given trial.

## 3 Simulations

**Fig. 2:**
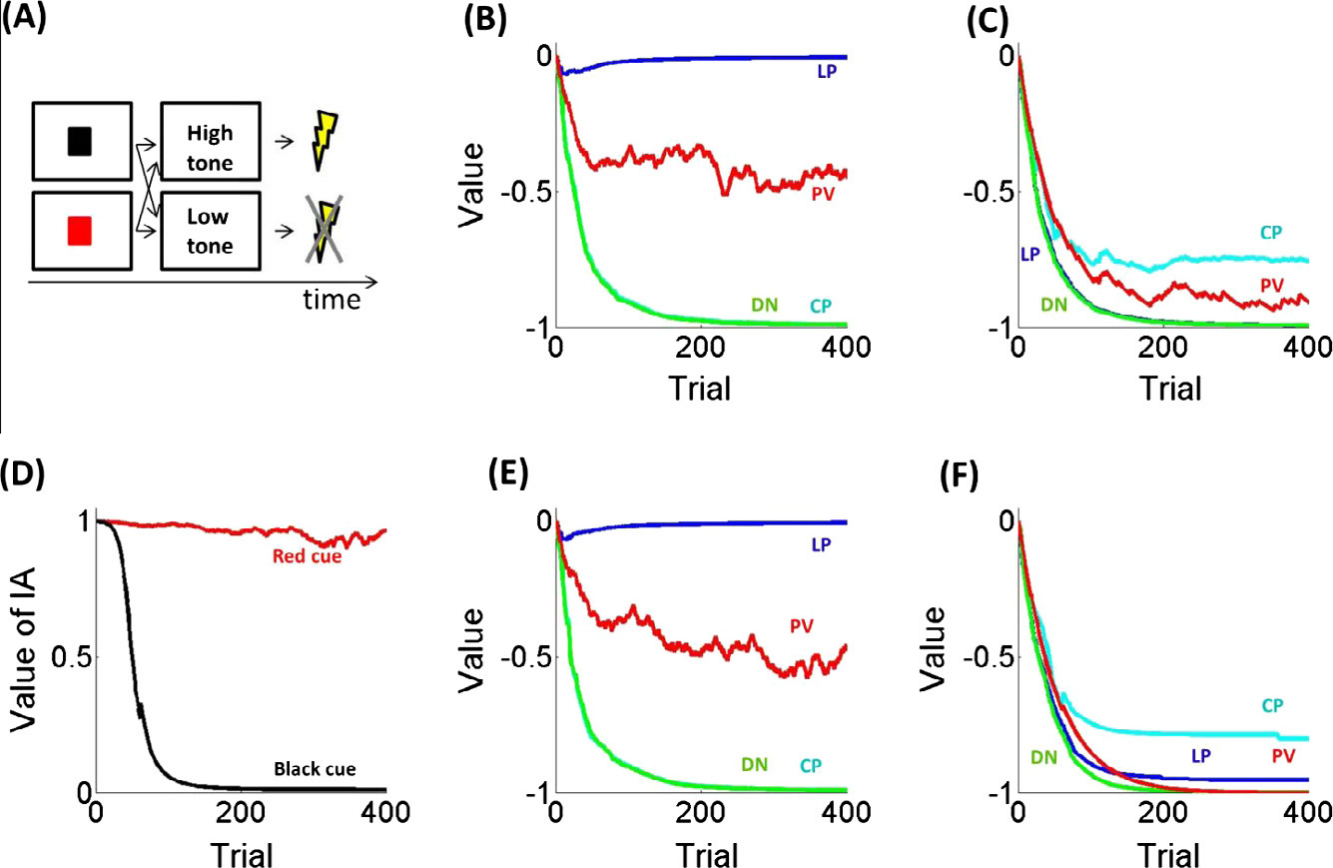
(A) Task used in simulations, in which for each trial a simulated rat is presented either a red or black visual cue followed either by a high auditory tone and shock or low tone and no shock, depending on the rat’s action. (B) Action value as computed by the goal-directed system (LP in blue: lever pressing; CP in cyan: chain pulling; DN in green: doing nothing) and Pavlovian value (PV in red) associated with the red cue (here LP always avoids shock, other actions never avoid shock) in the first simulation, in which the Pavlovian system is not allowed to influence behaviour. (C) Action value as computed by the goal-directed system and Pavlovian value associated with the black cue (here CP avoids shock 20% of the times, other actions never avoid shock) in the first simulation. (D) Instrumental ability (IA) for the red (here LP always avoids shock, other actions never avoid shock) and black cue (here CP avoids shock 20% of the times, other actions never avoid shock) in the second simulation in which the Pavlovian system is allowed to influence behaviour. (E) Action value as computed by the goal-directed system and Pavlovian value associated with the red cue in the second simulation. Colours are as in B. (F) Action value as computed by the goal-directed system and Pavlovian value associated with the black cue in the second simulation. Colours are as in B.

A specific version of the model was implemented in simulation experiments representing a scenario (Fig. 2A) wherein a simulated rat is presented with a chain and a lever. At every trial either a red or black visual cue appears followed, after few seconds, either by a high or low auditory tone. Here the high and low tones are associated respectively with delivery and omission of an electric shock stimulus with a negative value of one unit. In the time interval between the presentation of the visual cue and the tone, the rat is allowed to press the lever, pull the chain or do nothing. The action selected influences which auditory tone (either high or low) is presented and therefore whether punishment is delivered or not. At every trial, the most advantageous action depends on which visual cue is shown and hence, to minimise punishment, the rat has to learn the best action to perform with each visual cue (see below for contingencies used in simulations).

In relation to specific characteristics of the model used in simulations, PERC has two nodes, associated with the ‘red’ and ‘black’ visual cue, respectively. ACT has three nodes, associated with ‘lever pressing’, ‘chain pulling’, and ‘no action’, respectively. OUT has two nodes, associated with the ‘high’ and ‘low’ auditory tone, respectively. WM, GDP, PR and BEHAVIOUR have three nodes each, associated with the same actions as ACT, whereas GDV, PV, SC, GC and TSTD have one node each. In order to describe and test key characteristics of the model, we used five simulation experiments described in detail below.

### 3.1 Goal-directed control

The aim of the first simulation is to test the model’s ability to use goal-directed control to learn the correct actions in relation to different contexts. Task contingencies are as follows: when a red cue appears, lever pressing leads to a low tone and shock is always avoided while all other actions, namely chain pulling and doing nothing, lead to a high tone and shock. In the case where a black cue appears, chain pulling is better as shock is avoided 20% of times while it is always delivered by lever pressing or doing nothing. Here we test whether the goal-directed system can learn the correct actions associated with each of the two cues. In this simulation the goal-directed system alone is allowed to affect behaviour. Since goal-directed and Pavlovian processes are to some degree always co-activated in ecological circumstances, this condition is unrealistic; however, here we discuss it in order to better clarify how the goal-directed component works.

Data shown in Fig. 2B and C describe the value associated with each of the three actions. Pavlovian values associated to stimuli are also presented, although in this simulation by design they are not allowed to impact on behaviour. Results indicate that the agent is able to learn the correct policy both with the red (Fig. 2B) and black (Fig. 2C) cue. However, the asymptotic value related to the best action is higher with the former than the latter cue. This is consistent with the concept that asymptotic values represent the expected value of actions (Von Neumann & Morgenstern, 1944). Also, the asymptotic Pavlovian value is higher (i.e., less negative) with the red than the black cue, consistent with the fact that the Pavlovian value of each stimulus is proportional to the probability of punishment associated with that stimulus and is independent from the action performed. In relation to learning, the goal-directed subsystem learns two kinds of information, namely the causal associations between stimuli, actions, and outcomes and the outcome-value associations. Overall, these results show that the goal-directed subsystem can learn and choose consistent with models of prospective decision-making (Glimcher, 2004, Glimcher and Rustichini, 2004, Kahneman and Tversky, 1979).

### 3.2 Goal-directed/Pavlovian interaction

The aim of the second simulation is to analyse the relationship between Pavlovian and goal-directed mechanisms. Here, when a red cue is presented, lever pressing always avoids shock and shock is always delivered with other actions. When a black cue is presented, chain pulling leads to shock avoidance 20% of times and shock is always delivered with other actions. Contrary to the previous simulation, in this instance both goal-directed and Pavlovian subsystems are allowed to influence behaviour. In this and following simulations, the response triggered by the Pavlovian system is always ‘doing nothing’ to simulate a freezing response, and is never adaptive as it always leads to shock.

Results are reported in Fig. 2D and F showing the probability of the goal-directed system in the control of behaviour in front of the red (red line) and the black (black line) cues. At the beginning, behaviour is completely goal-directed in both contexts. Contingencies are unknown and hence actions are chosen randomly, leading often to shock and thus to a more negative Pavlovian value. However, at the same time knowledge about stimulus-action-outcome-value associations improves with learning and therefore with the red cue an effective action (i.e., lever pressing) is acquired leading to an increased Pavlovian value (Fig. 2D and E). By contrast, with the black cue the best action still leads to shock most of the times (although less than other actions) and therefore the Pavlovian value continues to decrease triggering an innate tendency to freezing corresponding to ‘do nothing’. Although this response is maladaptive, nonetheless it is maintained by a vicious circle whereby a negative Pavlovian value triggers a Pavlovian response followed by punishment that in turn decreases further the Pavlovian value.

These results are consistent with animal experiments showing that in some circumstances Pavlovian effects are detrimental for performance (Bolles, 1970, Guitart-Masip et al., 2014, Rigoli et al., 2012, Williams and Williams, 1969). Note that a key prediction stemming from this simulation is that the influence of Pavlovian over goal-directed control increases with the level of punishment expected, and this is consistent with empirical evidence. Fanselow and Bolles (1979) have shown that the probability of freezing correlates with punishment intensity, suggesting an enhanced Pavlovian strength with large punishment expectancy. However, a limit of this experiment is the lack of instrumental components. This limitation is addressed in another study (Bolles & Warren, 1965) showing that the probability of bar pressing to avoid shock decreases with shock intensity, suggesting that goal-directed behaviour (associated with bar pressing) is dominated by Pavlovian control with large punishment expectancy. This result is also consistent with a recent human study (Rigoli et al., 2012) where a stimulus moved on a computer screen and a button needed to be pressed when the stimulus was on a target. The colour of the target indicated whether an electric shock was delivered or not with a mistake and, in different trials, the stimulus could move fast or slow. For the fast condition, performance decreased when comparing shock versus no-shock trials. Crucially, this effect was enhanced in participants with poorer task performance, consistent with the idea that the Pavlovian influence dominated goal-directed behaviour in participants who expected more punishment (given their poor performance).

### 3.3 Modulatory role of specific controllability

We next explore effects of controllability related to specific contexts. Here the red cue leads to shock avoidance 20% of times independently of the action performed and the black cue leads to shock avoidance 20% of times with chain pulling and never with other actions. In this way, the red cue is associated with low controllability as no action is better than others, while the black cue is associated with a certain degree of controllability as one action is better than others. Crucially, the shock probability is equivalent with the red and black cues (in the latter case conditioned on the execution of the correct action). Here, we predict that different degrees of specific controllability influence the balance between goal-directed and Pavlovian activation.

**Fig. 3:**
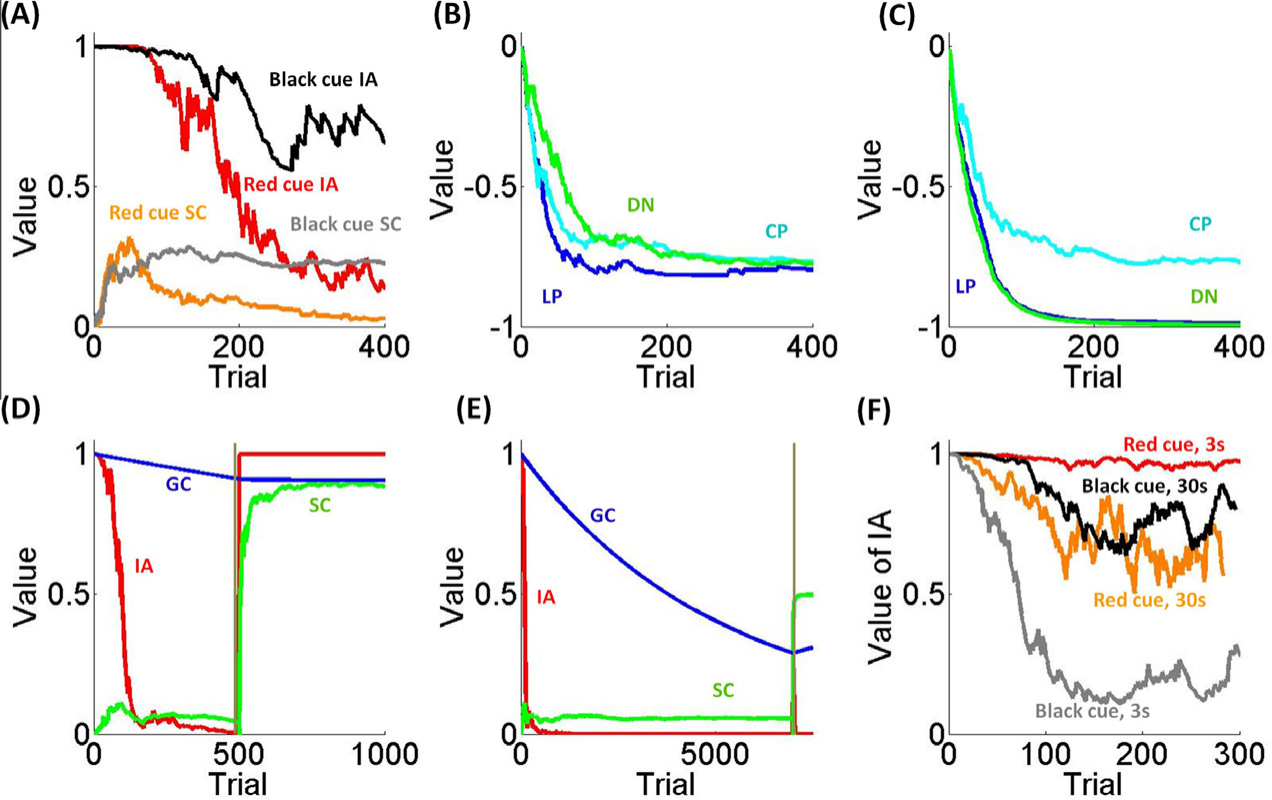
(A) Instrumental ability (IA) for the red (red line) and black (black line) cue and associated specific controllability (SC; orange line for red cue and grey line for black cue) in the third simulation. With the red cue, all actions avoid shock 20% of the time; with the black cue chain pulling avoids shock 20% of the time and other actions never avoid shock. (B) Action value as computed by the goal-directed system with the red cue in the third simulation (LP in blue: lever pressing; CP in cyan: chain pulling; DN in green: doing nothing). (C) Action value as computed by the goal-directed system with the black cue in the third simulation. (D) IA (in red), SC (in green) and general controllability (GC, in blue) for the first agent during simulation four (in trials 1–500, the red square was shown and shock occurred 90% of the times independently of the response; in trials 501–1000 the black square was shown and shock was avoided 90% of the times with chain pulling and always delivered with other actions). The grey bar represents the trial corresponding to the shift from red to black cue presentation. (E) IA, SC and GC (same colour as in D) for the second agent during simulation four (in trials 1–7000, the red square was shown and shock occurred 90% of the times independently of the response; in trials 7001–7500 the black square was shown and shock was avoided 90% of the times with chain pulling and always delivered with other actions). The grey bar represents the trial corresponding to the shift from red to black cue presentation. (F) IA for the red cue and 30 s delay from shock (red line), the red cue and 3 s delay from shock (orange line), the black cue and 30 s delay from shock (black line), and the black cue and 3 s delay from shock (grey line). For the red cue, lever pressing is followed by shock 20% of the times and shock is always delivered with other actions; for the black cue, chain pulling is followed by shock 40% of the times and shock is always delivered with other actions.

Fig. 3A shows that the probability that behaviour is goal-directed and the value of SC are asymptotically higher for the black than the red cue. Also, Fig. 3B and C shows that with the red cue action values remain roughly equal along trials, while with the black cue the value of the best action remains higher. These results show how the model implements a modulatory influence of specific controllability on the relative strength of goal-directed and Pavlovian control, as Pavlovian strength is inhibited when a given action is better than others (corresponding to higher controllability) and is boosted when action values are roughly equivalent (corresponding to lower controllability).

This is consistent with animal findings showing fear responses increase with uncontrollable, compared to controllable, shocks; even when punishment amount is equivalent in the two conditions (Desiderato and Newman, 1971, Mineka et al., 1984). However, some aspects of the simulation proposed here represent novel predictions that go beyond the available empirical data, and remain to be tested. Indeed, Mineka et al. (1984; see also Desiderato & Newman, 1971) trained two groups of rats with shock. While the first group could terminate shocks with an escape response, the second group received shock at the same time as the first group but could not affect punishment delivery. When exposed to the context where learning occurred, the second group of rats exhibited increased freezing. This experiment shows that Pavlovian responding is boosted by uncontrollable punishment, but leaves open the question of whether this impairs goal-directed behaviour, as we suggest in our simulation. In addition, previous experiments (Desiderato and Newman, 1971, Mineka et al., 1984) are in the context of shock escaping. Though our model makes similar predictions for both escape and avoidance contexts, these predictions remain to be empirically tested in avoidance.

### 3.4 Modulatory role of generalised controllability

In the model, controllability is factorized into two subcomponents, specific and generalised controllability. Specific controllability depends on the conditional probabilities of avoiding a punishment by acting in a given context while generalised controllability depends on the probability of avoiding punishments by acting independent from contexts. Here we test the role of generalised controllability, and whether manipulating this variable allows us to reproduce key empirical findings on learned helplessness.

We consider the same scenario as in previous simulations but now we group trials in two blocks. In all trials of the first block a red cue is presented and shock is delivered 90% of times independent of the action performed. In all trials of the second block a black cue is presented and shock is avoided 90% of times with chain pulling and 10% of times with other actions. We manipulated the amount of learning by comparing the performance of two agents characterised by the same parameters but experiencing a different number of trials in the first context (500 and 7000 trials for the first and second agent respectively). This is motivated by evidence indicating that learned helplessness effects emerge only after extensive experience in an uncontrollable environment (Seligman & Maier, 1967). Consistent with these findings, we expect the amount of learning in the uncontrollable context to influence the level of generalised controllability and in turn determine whether learned helplessness behaviour is exhibited in a novel context.

Agents’ performance is shown in Fig. 3D and E. In the first block, goal-directed strength and specific and generalised controllability decay for both agents, but generalised controllability decays more for the agent with extensive training. With a novel context, all quantities are reset except for generalised controllability so that the level of this variable remains high enough to elicit goal-directed control for the short-trained agent but not for the long-trained agent in which Pavlovian control is elicited also in the novel context. This manipulation reproduces data on learned helplessness showing that animals, after an extensive experience of uncontrollability, are unable to learn an effective instrumental response even in novel contexts that are potentially controllable (Maier and Seligman, 1976, Seligman and Maier, 1967).

### 3.5 Modulatory role of temporal and spatial threat distance

Temporal and spatial distance constitutes the other modulatory variable implemented in the model. We now test whether manipulating this variable influences behaviour. With the red cue shock is always avoided by lever pressing and never avoided with other actions. For the black cue shock is avoided 60% of times by chain pulling and never avoided with other actions. The time interval between the cue presentation and shock delivery randomly varies on two levels (3 and 30 s) across trials and is signalled during stimulus presentation. We expect that with the black cue (associated to higher goal-directed and Pavlovian values) behaviour is largely under goal-directed control though to a lesser extent when shock delivery is close in time, while with the red cue (associated to lower goal-directed and Pavlovian values) we expect goal-directed control to guide behaviour when the threat is far in time and Pavlovian control to guide behaviour when the threat is close in time.

These predictions are confirmed by results shown in Fig. 3F that is consistent with empirical evidence about the role of temporal and spatial threat distance played in aversive behaviour (Blanchard and Blanchard, 1989, Fanselow and Lester, 1988). Substantial evidence indicates that the probability of freezing decreases with shock delay (Fanselow & Lester, 1988). A similar role of threat distance is found in spatial contexts where the probability of freezing increases when a predator is close in space (Blanchard & Blanchard, 1989). These studies demonstrate that the Pavlovian strength, expressed by freezing behaviour, is boosted with short temporal and spatial distance. However, one limit of these studies is the lack of instrumental aspects, leaving open the question of whether Pavlovian control dominates goal-directed behaviour as threat distance diminishes. Evidence in favour of this hypothesis comes from a recent human study (Rigoli et al., 2012) where the impairing effect of a conditioned stimulus on instrumental behaviour emerged only in trials with a short temporal delay between the conditioned stimulus and the punishment.

## 4 Implications for neurobiology

Here we propose a connection between our model and neurobiology. In general, our implementation is consistent with the proposal that the aversive system is organised hierarchically in the brain along a rostro-caudal axis where different regions are preferentially recruited by specific levels of threat distance and are associated with distinct defensive reactions (Bravo-Rivera et al., 2014, Deakin and Graeff, 1991, Fanselow, 1994, McNaughton and Corr, 2004). Evidence shows that distal or potential threats recruit preferentially rostral areas such as dorsolateral prefrontal cortex (DLPFC), orbitofrontal cortex (OFC), hippocampus and ventromedial prefrontal cortex (vmPFC), whereas amygdala and periaqueductal grey (PAG) play a central role in processing proximal threats (Blanchard and Blanchard, 1989, Deakin and Graeff, 1991, Fanselow, 1994, Graeff, 2004, Keay and Bandler, 2001, Keay and Bandler, 2002, McNaughton and Corr, 2004). Our model connects the neural hierarchy to the distinction between goal-directed and Pavlovian forms of control.

More specifically, each subsystem in the model can be mapped to a specific brain circuit, with PERC implemented in sensory cortical and subcortical areas and ACT related to regions involved in (abstract) motor representations such as the supplemental motor area and the premotor cortex (Rizzolatti et al., 1988). A role in ACT might be played also by the caudate nucleus and the putamen of the basal ganglia (corresponding to the dorsolateral and dorsomedial striatum in rodents, respectively), which are involved in instrumental, but not Pavlovian, action selection (Pennartz et al., 2011, Yin et al., 2008). OUT, associated with mental simulation of future sensory states, might recruit regions involved in processing abstract state representations such as (i) the hippocampus, where cells encoding the spatial position of an animal (the so-called place cells) sweep forward at decision points and can code future trajectories when the animal rests or sleeps, consistent with planning and the mental simulation of possible future positions (Diba and Buzsáki, 2007, Johnson and Redish, 2007, Pezzulo et al., 2013, Pezzulo et al., 2014, Pfeiffer and Foster, 2013, Wikenheiser and Redish, 2015), (ii) more broadly, the medio-temporal lobe, a region involved in episodic memory and in representing abstract categories (Hassabis and Maguire, 2007, Squire et al., 2004). Based on evidence highlighting a role for OFC in representing specifically outcome (but not action) value, one possibility is that this region processes GDV, corresponding to the value of future states (Schoenbaum, Takahashi, Liu, & McDannald, 2011). Substantial evidence has indicated a central role of DLPFC in executive functions, and specifically in working memory, corresponding to WM in our model, and choice process, corresponding to GDP (Gold and Shadlen, 2007, Koechlin and Summerfield, 2007, Miller and Cohen, 2001, Stoianov et al., 2015).

In relation with Pavlovian mechanisms, unconditioned fight/flight reactions and non-opioid analgesia are regulated by lateral PAG (lPAG) and hypothalamus (Keay and Bandler, 2001, Keay and Bandler, 2002), and conditioned freezing responses and opioid analgesia by ventro-lateral PAG (vlPAG; Fanselow, 1994, Keay and Bandler, 2001, Keay and Bandler, 2002). In addition, amygdala plays a central role in storing Pavlovian representations (Cardinal et al., 2002, Davis, 1992), with basolateral nuclei encoding conditioned-unconditioned stimulus associations (Amorapanth et al., 2000, Choi et al., 2010, Lázaro-Muñoz et al., 2010) and central extended nuclei controlling different aspects of conditioned responses such as motor reactions, opioid-mediated analgesia (through connections with vlPAG), hormonal and autonomic reactions (through hypothalamic connections), and vigilance associations (Amorapanth et al., 2000, Choi et al., 2010, Lázaro-Muñoz et al., 2010). Another important role is played by the ventral striatum of the basal ganglia, which processes Pavlovian values associated with conditioned stimuli (Cardinal et al., 2002, Yin et al., 2008).

Evidence indicates that an increased response in the dorsal raphé nuclei (DRN) elicits learned helplessness behaviour, while activation in vmPFC inhibits such behaviour (Amat et al., 2005, Maier and Watkins, 2005). A possibility is that GC, representing a generalised belief about controllability, is reflected in the firing rate of DRN neurons, while SC, indicating a controllability belief related to the current context, might instead be processed in vmPFC. This is consistent with the finding that vmPFC activity during decision-making correlates with the value difference across options (Boorman et al., 2009, Hunt et al., 2012, Strait et al., 2014), a signal similar to SC.

It has been reported that processing of emotional, compared to neutral, stimuli recruits amygdala directly via thalamo, bypassing the cortex (Vuilleumier & Driver, 2007). It is possible that such neural pathway is modulated by the temporal and spatial threat distance in such a way that it is preferentially recruited during perception of proximal dangers. Another aspect relevant to threat distance is that physical contact with danger directly stimulates the nociceptive, tactile and proprioceptive receptors of PAG (Keay and Bandler, 2001, Keay and Bandler, 2002).

Learning corresponds to changing synaptic strength. A Hebbian form of learning characterises acquisition of state-action-outcome contingencies and is linked to glutammatergic and gabaergic neural mechanisms (Izquierdo & McGaugh, 2000). A central role in value learning is attributed to dopamine based on evidence that response of this neurotransmitter reflects a reinforcer prediction error signal, both in instrumental (Berridge, 2007, Hollerman and Schultz, 1998) and Pavlovian contexts (Schultz et al., 1997, Wenzel et al., 2014). A key role has been proposed also for serotonin whose function would be opponent to dopamine, though evidence is mixed (Boureau & Dayan, 2011). Serotonin has also been linked to controllability and specifically to activity in DRN, a major serotoninergic hub in the brain (Maier & Watkins, 2005). A possibility is that this neurotransmitter is involved in learning a general form of controllability, which is independent of the current context. This might suggest that the opponency between dopamine and serotonin might be only partial, being the former linked with learning values attached to specific contexts and the latter linked with learning a controllability belief independent of contexts. This hypothesis remains to be tested in future research.

## 5 Discussion

We propose a computational model of aversion based on a goal-directed/Pavlovian interaction wherein controllability and threat distance occupy an important modulatory role by influencing the relative strength of the two controllers. The integration of multifaceted motivational mechanisms is an important aspect of this proposal given that most previous theories have considered only partial components of aversion. Indeed, associative-learning models have largely focused on reactive Pavlovian behaviour (Blanchard and Blanchard, 1989, Deakin and Graeff, 1991, Dinsmoor, 2001, Fanselow, 1994, Fanselow and Lester, 1988, Graeff, 2004, McNaughton and Corr, 2004), whereas most normative decision-making theories implicitly assume goal-directed control alone (Glimcher, 2004, Kahneman and Tversky, 1979).

Our model is inspired by recent proposals that view behaviour as guided by a multicontroller system that integrates instrumental and Pavlovian components (Dayan et al., 2006, Guitart-Masip et al., 2014, Moutoussis et al., 2008, Rigoli et al., 2012). We also stress the link with a set of neural network models that combine reinforcement learning principles within a biologically plausible implementation. This permits us to connect model architectures and computations to neural structures and functions, respectively (Frank et al., 2004, Miller and Cohen, 2001, Reynolds and O’Reilly, 2009).

Though debate remains regarding the precise mechanisms underlying the Pavlovian/goal-directed interactions, we assume these systems work in parallel as each performs its specific computations at the same time as the other. An alternative possibility is that a meta-decision process allocates resources to one or the other controller before they perform their specific computations. Future research is needed to elucidate this point.

There is strong evidence that the two systems interact at different levels. Here we focus on competition at the motor level based on evidence that (i) Pavlovian stimuli can inhibit a general motor reactivity (Gray, 1987, Gray and McNaughton, 2000), (ii) non-specific Pavlovian responses such as trembling can impair the precision of motor commands (Rigoli et al., 2012) (iii) specific Pavlovian motor actions can influence the execution of incompatible instrumental behaviour (Morse, Mead, & Kelleher, 1967). Other levels are involved in the goal-directed/Pavlovian interaction as fearful stimuli can exert a Pavlovian influence on executive functions usually associated with goal-directed control, for instance by speeding and biasing attentional processes (Eysenck, Derakshan, Santos, & Calvo, 2007). Another set of interaction effects occurs at the level of value computation, as in Pavlovian–instrumental transfer (PIT) and conditioned suppression where a Pavlovian stimulus increases (or decreases) the motivation to approach (or avoid) other appetitive (or aversive) outcomes especially those also predicted by the same Pavlovian stimulus as in specific PIT (Bray et al., 2008, Campese et al., 2013, Campese et al., 2014, Dickinson and Pearce, 1977, Holland, 2004, Overmier et al., 1971, Rescorla and Solomon, 1967).

Here we focus on goal-directed–Pavlovian interactions, though models of instrumental control include also the so-called habitual system, which is based on stimulus–response associations learned through the history of reinforcement (Adams, 1982; Colwill and Rescorla, 1988, Daw et al., 2005) and is thought to overwhelm goal-directed control in simple environments and after extensive training (Dolan & Dayan, 2013). It is important to stress that, despite some notable exceptions (e.g., Holland, 2004, Rigoli et al., 2012), most of the data available on aversion do not distinguish between goal-directed and habitual control. Future research is needed to clarify whether the influence of the Pavlovian system changes with goal-directed compared to habitual control, though we note that some empirical evidence suggests Pavlovian effects might even be enhanced in the latter case (Holland, 2004, Rigoli et al., 2012).

In keeping with a large body of empirical evidence, in our model a key role is attributed to threat distance and controllability. The importance of threat distance has been stressed in previous models, but here we extend this idea by arguing this variable not only influences which defensive reaction is exhibited but also which form of control, Pavlovian or goal-directed, is activated. Specifically, our model proposes that the Pavlovian strength is boosted as threat distance decreases. A similar point is proposed with respect to controllability together with the distinction of different hierarchical levels that represent this variable, including contextual-dependent and contextual-independent components. The inclusion of two components that are organised hierarchically can account for different empirical phenomena, reconciling competing theories on the role controllability (Maier and Seligman, 1976, Mineka et al., 1984, Seligman and Maier, 1967). Indeed a specific controllability factor can account for a finding that fear responses increase with uncontrollable, compared to controllable, punishments (Desiderato and Newman, 1971, Mineka et al., 1984). A general controllability factor accounts for evidence that uncontrollability effects are generalised to new contexts by impairing instrumental learning (Maier and Seligman, 1976, Seligman and Maier, 1967).

Fear and anxiety are emotional responses favoured by evolution for their efficacy in dealing with danger. An influential perspective suggests that these are two separate emotions as controlled by specific psychological and neural systems and triggered by specific aversive conditions, with threat distance determining which of the two is activated (Blanchard and Blanchard, 1989, Davis et al., 2009, Deakin and Graeff, 1991, Fanselow, 1994, Fanselow and Lester, 1988, Graeff, 2004, LeDoux and Gorman, 2014, McNaughton and Corr, 2004). Specifically, fear would correspond to a set of fight/flight reactions elicited by proximal and certain threats, whereas anxiety would be characterised by more complex processes such as worrying tendencies elicited by distal and uncertain threats. In our scheme, fear and anxiety are viewed as parts of a continuum which describes the goal-directed/Pavlovian relative weight, with controllability and threat distance determining the current position within the continuum. One extreme of the continuum corresponds to a state of mild anxiety, characterised by the belief that the threat is still far and controllable. Here, goal-directed planning prevails and the influence of Pavlovian behaviour is negligible. As one moves towards the other extreme, the perception of threat distance and controllability decreases, anxiety enhances, and the Pavlovian influence emerges. In this condition of increased anxiety, goal-directed planning is still important but Pavlovian reactions, such as an automatic attention towards threat and an increased physiological response (Eysenck et al., 2007), are also manifested. Note that such state of elevated anxiety is characterised by an intermediate level of controllability and threat distance. As we approach the other extreme of the continuum, controllability and threat distance diminish, goal-directed control is disrupted and fight/flight/freezing Pavlovian reactions dominate, a condition associated to fear. Note that, in this view, fear and anxiety are not qualitatively different emotions like in some other theories (Davis et al., 2009, Deakin and Graeff, 1991, Fanselow, 1994, Fanselow and Lester, 1988, McNaughton and Corr, 2004), but share common Pavlovian processes (though there might be aspects of the Pavlovian response which might be activated only during fear and not anxiety and vice versa). In addition, the transition from anxiety to fear is graded. This perspective suggests that one of the key factors of pathological anxiety might be a bias towards perceiving decreased threat distance and controllability. This would lead to an exaggerated anxiety response despite the true levels of controllability and threat distance are high, and to a fear response in conditions where an anxious response would be appropriate. Our view can be conceived as a formalisation and extension of a previous influential theory which proposes that the key dysfunction in exaggerated anxiety is an increased anxiety response with distal threats but not proximal threats (Mathews & Mackintosh, 1998).

Our model is based on some arbitrary assumptions and simplifications. One of these is that goal-directed planning follows a serial process by which different actions are simulated sequentially. This might be too simplistic, though the idea that executive functions require serial computations is supported by some data (Miller & Cohen, 2001). Other assumptions are about the choice process, as we assume that even after extensive training an agent exhibits randomness in choice due to a softmax decision rule, again based on empirical support (Daw, O’Doherty, Dayan, Seymour, & Dolan, 2006). A further simplification is in the use of a fixed learning rate, at variance with evidence that this parameter depends on uncertainty or environmental volatility (Behrens et al., 2007, Pezzulo et al., 2013). One possibility is that uncertainty about the values encoded by the goal-directed and Pavlovian control might also modulate the relative strength of each controller (Daw et al., 2005, Pezzulo et al., 2015, Pezzulo et al., 2013). The Pavlovian subsystem is implemented as a set of stimulus–response associations learned through punishment experience, though this is likely to be an oversimplification given evidence that Pavlovian responses are also elicited by stimulus-outcome associations (Dickinson & Balleine, 2002). However, it is unclear in which circumstances Pavlovian mechanisms are under the control of stimulus–response and stimulus-outcome associations and how these different representations interact.

Our model can deal with problems having multi-steps temporal horizons, though these scenarios are not considered in our simulations. A limit of the model is that it works with simple problems with a small state space and with relatively short temporal horizons. A fundamental issue arising from problems with large state space is that computing the optimal policy becomes computationally expensive or intractable, and, to account for this, approximations such as sampling methods are often adopted (Pezzulo et al., 2013). A way to implement these approximations in our model could be to set an order for policy/action simulation during goal-directed planning, implemented through the pattern of inhibitory connections among policy/action nodes.

## 6 Conclusions

We propose a computational model of aversion that takes into account different kinds of computations and their complex interaction and integrate them in a broad and unifying picture. We believe this might provide a useful reference for empirical research as can help generate new hypotheses and guide the setting of priorities on research questions. Moreover, given the ubiquity and relevance of aversive conditions in everyday contexts, the model can help a better understanding of important aspects in clinical and intervention settings, and here we provide an example in relation with negative emotions.

## Figures and Tables

**Table 1:** Parameters used in the simulations.

Parameter	Sim. 1	Sim. 2	Sim. 3	Sim. 4	Sim. 5
$\beta_{0}$	80	80	80	−120	80
$\beta_{\text{GDP}}$	30	30	30	30	30
$\beta_{\text{PR}}$	0	100	100	100	100
$\beta_{\text{SC}}$	0	0	200	200	0
$\beta_{\text{GC}}$	0	0	0	200	0
$\beta_{\text{TSD}}$	0	0	0	0	1
$\alpha_{\text{M}1}$	1	1	1	1	1
$\alpha_{\text{GDV}}$	0.02	0.02	0.02	0.02	0.02
$\alpha_{\text{PV}}$	0.02	0.02	0.02	0.02	0.02
$\alpha_{\text{GC}}$	0	0	0	0.0002	0
n. of trials	1200	1200	1200	1000/7500	1200

## Appendix A

In this section, the algorithm implemented by the model is described in detail. The model is composed of layers grouped in different subsystems. The first subsystem is the goal-directed controller, composed by ACT, OUT, GDV, WM, and GDP. For implementations involving multi-step horizons, ACT, OUT and GDV are replicated for each time step and POL and GDV-SUM are included. Each ACT (step function) neuron corresponds to a simulated action; Each OUT (linear function) neuron corresponds to an expected outcome; GDV has only a (linear function) neuron, which corresponds to the value of the currently simulated action; WM encodes the memorised action values, and has the same number of neurons as ACT (although, in this case, they are linear function neurons); GDP encodes the selected action, having the same number of (linear function) neurons as WM. In multi-steps horizon problems, POL contains as many (step function) nodes as the number of combinations of node activations within the different ACTs along time and GDV-SUM includes a (linear function) neuron.

For one-step temporal horizon implementations, the dynamic of the goal-directed subsystem is as follows. At the beginning of each trial, all neurons have a null activation. A stimulus *i* is detected in the environment activating the corresponding PERC(*i*) neuron which sends an output signal equal to one to all ACT nodes. ACT nodes are step function neurons whose activity is equal to zero if the corresponding input is equal or smaller than zero, and equal to one if the corresponding input is larger than zero. Each ACT neuron sends an inhibitory output equal to minus one to all other neurons in ACT with a larger index. For this reason, although PERC(*i*) excites all ACT neurons, only the first one is activated, while all other neurons are inhibited by the first one. PERC–ACT–OUT connections are represented by a weight matrix M(*I*, *J*, *Z*), where *I*, *J* and *Z* are the number of nodes in PERC, ACT and OUT, respectively. When the first ACT neuron is activated, an ACT–PERC combination (*i*, 1) activates the vector OUT(:) = M(*i*, *1*, :)/sum(M(*i*, 1, :)). The OUT vector is multiplied by the OUT–GDV connection vector, and the result is the scalar activation of the GDV neuron. The GDV value is then multiplied by the ACT vector, and the resulting vector sums up to the initial WM zero vector. After this process, the first neuron of WM has an activation which is equal to the GDV value, while all other neurons continue to have a null activation. At this point, the goal-directed process continues in a recursive way. Indeed, WM has an inhibitory connection with ACT. In particular, the *x*th WM neuron sends an output to the *x*th ACT neuron, so as, if WM(*x*) > 0, then ACT(*x*) = 0. Since after the first cycle WM(1) > 0, then ACT(1) neuron is inhibited by WM(1). For this reason, now the second neuron in ACT is no more inhibited by the first one (which is now inhibited by WM). At the same time, all other neurons are inhibited by the second ACT neuron. At this point, the computations are repeated as described before, until all ACT neurons have been activated. At the beginning of every cycle, all neural activations decay, except those related to PERC and WM. In relation to the latter layer, every time the resulting vector of the multiplication between GDV and ACT is computed, it sums up to the WM vector of the previous cycle, and the resulting vector is the new WM vector. Once all WM neurons, which represent the action values, have been computed, one of the GDP neurons is activated. The index of this neuron is extracted from a distribution whose elements have a probability equal to the corresponding normalised action values. The activation level of the GDP neuron corresponds to the activation of the highest activation neuron in WM, even when the latter neuron and the activated GDP neuron have a different index.

For multi-steps horizon implementations, the *i* input recruits the PERC(*i*) neuron which sends an output signal equal to one to all POL nodes which are step function neurons whose activity is equal to zero if the corresponding input is equal or smaller than zero, and equal to one if the corresponding input is larger than zero. Each POL neuron sends an inhibitory output equal to minus one to all other neurons in POL with a larger index. For this reason, although PERC(*i*) excites all POL neurons, only the first one is activated, while all other neurons are inhibited by the first one. An activation of the first POL node induces activity in a certain combination of nodes within the different ACT layers along time. The active node *j****_1_*** of the first (in temporal order) ACT and of the active node *i* of PERC activate the node vector of the first OUT(:) = M(*i*, *j****_1_***, :)/sum(M(*i*, *j****_1_***, *:*)). The first OUT vector is multiplied by the OUT–GDV connection vector, and the result is the scalar activation of the first GDV. Next, the active node *j****_2_*** of the second ACT and the vector of the first OUT activate the vector of the second OUT in which activity of each node corresponds to OUT(z**_2_**) = sum(M(:, *j****_2_***, z**_2_**)/sum(M(:, *j****_1_***, :)). The vector of the second OUT is multiplied by the OUT–GDV connection vector, and the result is the scalar activation of the second GDV. This process is repeated along time until the last GDV is computed and all GDVs are summed up in GDV-SUM (at this stage it is possible to implement temporal discounting by multiplying each GDV by a corresponding discounting factor), which is next recorded in WM. After the first POL node is evaluated, planning follows the same dynamic as that described above for the one-step horizon implementation involving WM and ACT, except that now POL plays the role of ACT. Similarly, each time a new POL node is activated, the policy evaluation process follows the process described above for the one-step horizon implementation.

The second subsystem is the Pavlovian controller, whose layers are PV and PR. The former is composed of a (linear function) neuron, whose activity depends on PERC vector multiplied by the PERC–PV connection vector. PR is composed of the same number of neurons as ACT, but in this case neurons are linear function ones. Their activation corresponds to the product of PV and the PV–PR connection vector. All PV–PR vector neurons have value equal to zero except the one corresponding to the innate reaction with a value of one. The third subsystem is related to modulator variables including SC, GC and TSTD each represented by a linear function neuron.

Once GDP, PR, SC, GC and TSTD have been computed, the IA neuron activation is calculated. IA neuron is a sigmoid function neuron whose value is computed as follows:

$$\text{IA}=\frac{1}{1+\exp(-0.1(\beta_{0}+\beta_{\text{GDP}}\text{GDP}(\text{s})+\beta_{\text{PR}}\text{PR}+\beta_{\text{SC}}\text{SC}+\beta_{\text{GC}}\text{GC}+\beta_{\text{TSD}}\text{TSTD}}$$

where GDP(s) corresponds to the active GDP neuron, PR corresponds to the PR neuron associated with the Pavlovian innate reaction, and *β* parameters represent weights. Finally, BEHAVIOUR depends on which number is extracted from a binomial distribution whose parameter is IA. If the extracted number is 1, then BEHAVIOUR = GDP(s). If the extracted number is zero, BEHAVIOUR depends on PR(s). When PR(s) < 0, then BEHAVIOUR = PR(s); when PR(s) = 0, then BEHAVIOUR corresponds to a random action.

Once an outcome (OUTCOME) associated with a scalar hedonic value *V* ⩽ 0 is collected, M (i.e., the PERC–ACT–OUT connection matrix), is updated by a learning rate (α**_M1_**) added to the weight M(STIMULUS, OUTCOME, BEHAVIOUR). The OUT–GDV(OUTCOME) and PERC–PV(STIMULUS) connection weights and the GC value are updated according to a delta rule by summing a prediction error multiplied by a learning rate (respectively α**_GDV_**, α**_PV_** and α**_GC_**) to the previous value. The prediction error depends on *V* both for the OUT–GDV(OUTCOME) weight and the PERC–PV(STIMULUS) weight, and on SC for GC.

For the simulations, initial weights of the M matrix are set to one and other weights to zero. Initial GC value is set to one and the temperature parameter of the softmax function used to choose the action in GDP is assigned a value of one. Parameter values used in the simulations are reported in Table 1.
